# Effect of Chemically Treated Kenaf Fibre on Mechanical and Thermal Properties of PLA Composites Prepared through Fused Deposition Modeling (FDM)

**DOI:** 10.3390/polym13193299

**Published:** 2021-09-27

**Authors:** Aida Haryati Jamadi, Nadlene Razali, Michal Petrů, Mastura Mohammad Taha, Noryani Muhammad, Rushdan Ahmad Ilyas

**Affiliations:** 1 Fakulti Kejuruteraan Mekanikal, Universiti Teknikal Malaysia Melaka, Melaka 76100, Malaysia; aidaaaharyati@gmail.com (A.H.J.); noryani@utem.edu.my (N.M.); 2 Centre for Advanced Research on Energy, Universiti Teknikal Malaysia Melaka, Melaka 76100, Malaysia; mastura.taha@utem.edu.my; 3 Faculty of Mechanical Engineering, Technical University of Liberec, Studentská 2, 46117 Liberec, Czech Republic; 4 Fakulti Teknologi Kejuruteraan Mekanikal dan Pembuatan, Universiti Teknikal Malaysia Melaka, Melaka 76100, Malaysia; 5 School of Chemical and Energy Engineering, Faculty of Engineering, Universiti Teknologi Malaysia, Johor Bahru 81310, Malaysia; ahmadilyas@utm.my; 6 Centre for Advanced Composite Materials (CACM), Universiti Teknologi Malaysia, Johor Bahru 81310, Malaysia

**Keywords:** kenaf fibre, fibre treatment, mechanical properties, thermal properties, Fused Deposition Modelling (FDM)

## Abstract

Natural fibre as a reinforcing agent has been widely used in many industries in this era. However, the reinforcing agent devotes a better strength when embedded with a polymer matrix. Nevertheless, the characteristic of natural fibre and polymer matrix are in contrast, as natural fibre is hydrophilic, while polymer is hydrophobic in nature. Natural fibre is highly hydrophilic due to the presence of a hydroxyl group (-OH), while polymer matrix has an inherent hydrophobic characteristic which repels water. This issue has been fixed by modifying the natural fibre’s surface using a chemical treatment combining an alkaline treatment and a silane coupling agent. This modifying process of natural fibre might reduce the attraction of water and moisture content and increase natural fibre surface roughness, which improves the interfacial bonding between these two phases. In this paper, the effect of alkaline and silane treatment has been proven by performing the mechanical test, Scanning Electron Micrograph (SEM), and Fourier Transform Infrared spectrometry (FTIR) to observe the surface structure. The chemical compositions and thermal properties of the composites have been obtained by performing Differential Scanning Calorimetry (DSC) and Thermogravimetric Analysis (TGA) tests. 1.0% silane treatment displayed better strength performance as compared to other composites, which was proven by performing Scanning Electron Micrograph (SEM). The assumption is that by enduring chemical treatment, kenaf fibre composites could develop high performance in industry applications.

## 1. Introduction

Nowadays, attention of engineers and professionals has been triggered regarding the increased consumption of petroleum and the depletion of these sources. In addition, the emission of harmful gas into the environment and the greenhouse effect during incineration produced an alternative in the development and sustainability of natural polymer composites [1,2]. Aerospace, automotive, and construction industries have widely used advanced polymer composites, which contain carbon and glass fibre as the primary materials [3]. It was found that these primary materials are hardly reusable and reutilised [3]. Therefore, natural fibre has been introduced to replace the consumption of petroleum-based and synthetic fibres. Other than that, the characteristics between natural fibres and synthetic fibres are quite similar, such as low density, high stiffness, and good mechanical properties [1]. In comparison to the characteristics of other fibres such as synthetic, glass, and carbon, natural fibre [4] shows an advantage in biodegradability, renewability, non-toxicity, CO_2_ neutral life cycle, degradablity, sustainability, and environmentally friendliness [2,3,5,6,7,8,9,10]. Table 1 shows the properties of natural fibre compared to other synthetic fibre. 

Next, besides greenhouse protection, advantages of natural fibre properties include less machine wear during processing, no health hazards, a high degree of flexibility, low cost, its light weight, ease of separation, fracture resistance, high sound absorption, less equipment abrasion, low respiratory irritation, vibration damping, enhanced energy recovery, and good thermal insulation [1,3,5,6,7,9,16,17], all of which encourage researchers and industrial engineers to use natural fibre as a main material in development [4]. Mechanical characteristics of natural fibre have high specific benefits such as stiffness, impact resistance, modulus, strength, and durability [9,16,18]. These characteristics of natural fibres are well utilised in industrial product applications such as windows, frames, door panels, railroad sleepers, furniture, automotive dashboards and brake linings, shelves, egg-boxes, electronics packaging, textiles, and also building material applications [2,7]. Plant, animal, and mineral fibres are classified under the natural fibres division [5]. The most famous natural fibres used in the industrial platform is plant fibre, also known as lignocellulosic or cellulosic fibre. Pineapple (PALF), kenaf, coir, sisal, jute, hemp, flax, ramie and wood are examples of natural fibres that are popular in the applications platform and are usually used as reinforcing agents when combined with a biodegradable or non-biodegradable polymer matrix [2,3,7,19]. Kenaf fibre (KF), also known as *Hibiscus cannibus* L. [2,10], is one of the excellent substitutes for synthetic fibres [7]; it is also bio-based and available in tropical countries such as Thailand and Malaysia [20]. The kenaf plant demand is high because it grows rapidly which sustains its availability, and it is also low in cost [2,7,21]. Kenaf bast is a popular fibre used by researchers and scientists due to its outstanding mechanical properties such as flexural and tensile strength [10]. In addition to kenaf bast, flax and hemp are also popular natural fibre choices. [16]. As the pollution rate and environmental conditions consistently worsen, natural fibre, as a common material used in biodegradable products has been proposed by researchers, scientists, and engineers for application in industrial platforms such as the automotive, aerospace, aircraft, marine, and packaging industries [5,8,17]. Natural fibres are lightweight and have good mechanical properties [22]. Currently, most industries have resorted to the use of plastics [23,24]. In advanced applications, thermoplastic polymers are widely used, but due to their disadvantages which are lower in thermal stability and strength, some applications might not be applicable [24]. One of the renewable and biodegradable base polymers in the polyester group is Polylactic Acid (PLA) [21,22,25,26], which emits less CO_2_ gas and shows that this material is not harmful to the greenhouse, humans, and animals [22]. A number of reactive groups, which mostly contain biopolymers offer excellent composite blends between natural fibres and matrix polymers [26].

The hydrophilic property is innate in natural fibres such as kenaf. This property states that due to the presence of a hydroxyl group (-OH) in their cellulose structure as (-OH) exists in natural fibre’s structure, the moisture content might increase gradually [1,4,7,16,27]. This moisture content effect can cause swelling in structure and instability of dimension and also can lead to cracking [10]. This characteristic also has a disadvantage, which is that it could affect the adhesion bonding between the fibre and a polymer matrix, thereby producing unsatisfactory test results [16,19]. It could, in fact, contribute to low mechanical properties, low strength and short life span due to low interphase bonding between the fibre and polymer matrix [5,6]. Regarding these hydrophilic and hydrophobic issues, researchers state that one solution that might work is enhancement adhesion bonding between two phases and also improving the mechanical properties of the biocomposites by applying a surface modification [28]. M. Shirazi et al. (2019) stated that alkaline treatment was the most popular treatment in the surface modification process, and required the immersion of natural fibre in a sodium hydroxide solution within a certain timeframe [20,21]. This process could also remove impurities such as wax and oil from natural fibres and increase its surface roughness [2,9,21,24,26]. Some research experiments suggest surface modification will endure the alkaline treatment and silane coupling agent. In order to improve the wettability of natural fibres by polymer matrix and promote interfacial bonding, methods such as applying a coupling agent are used [8]. Silane is an example of a coupling agent in surface modification that shows excellent treatment, and importantly can improve interlocking adhesion between the fibre and polymer matrix better than other treatments [1,29,30]. It also interacts with chemical bonds of natural fibre and polymer matrix.

M. Asim et. al. (2016) conducted an experiment regarding surface treatment between kenaf fibre and PALF composites. In this experiment, data were collected from four different parameters: untreated fibre, alkaline-treated fibre, alkaline–silane-treated fibre, and silane-treated fibre. From the researchers’ observation, by enduring alkaline treatment, all the impurities in fibre can be removed completely, depending on alkali concentration and soaking time. By performing surface treatment, enhancement of strength in composites could occur [7].

Overall, the production of natural fibre is a new issue that has been introduced by many researchers. Natural fibre-reinforced polymer biocomposites using environmentally friendly FDM technology has attracted many industries and researchers. The implementation of natural fibres in the filament of FDM to replace the current fillers has attracted many competitors and market platforms [31]. The most popular polymer that acts as the main material in FDM is acrylonitrile butadiene styrene (ABS). However, the use of a thermoplastics polymer as the main material for FDM is still not recommended. The important elements of a polymer are its mechanical properties, which are strength and stiffness. As previously stated, the mechanical aspects of many bio-based polymers have been investigated to enhance the technology of FDM. Acrylonitrile butadiene styrene (ABS) and polylactic acid (PLA) are popular because they are stable. The most frequent thermoplastic that had been produced in this technology is PLA. The advantages of using PLA are that it is recyclable, biodegradable and has a temperature of 145–160 °C [32]. PLA is one of the biopolymers that is obtained from the fermentation of the recyclable product and has good mechanical properties such as tensile strength and low thermal stability that prevents crystallisation [33]. As previously stated, PLA is getting attention as a biodegradable and renewable plastic. It is also environmentally friendly, and the study of natural fibres such as hemp and kenaf as reinforcement combined with PLA using a standard method has also been done [25]. The fibre loading optimisation and also the chemical treatment of the reinforcement can affect the mechanical properties of the product. Therefore, the natural fibre that combines with the PLA is firm and requires dried feedstock and storage [25].

In this paper, the authors studied the treatment of kenaf fibre with a NaOH concentration of 6% for 24 h, followed by the chemical treatment of a silane coupling agent with three different concentrations, 0.5%, 1%, and 2%, respectively for 3 h to modify the surface characterisation of the natural fibre. This paper aims to investigate the effect of chemically treated kenaf fibres on mechanical and thermal properties of kenaf fibre-reinforced PLA composites. The effect of alkaline and silane treatment for surface modification has also been studied.

## 2. Materials and Methods

### 2.1. Materials

Kenaf fibre powder (unsieved) was supplied locally from Lembaga Kenaf dan Tembakau Negara (LKTN) before being treated and mixed with Poly Lactic Acid (PLA) pellets. Poly Lactic Acid (PLA), Silane (Aminopropyltriethoxysilane Agent) was obtained from Mecha Solve Engineering (Selangor, Malaysia).

### 2.2. Methodology

#### 2.2.1. Alkaline Treatment

In this experiment, kenaf fibre powder with random size (unsieved) within 100–650 μ were treated with alkaline treatment. The kenaf fibres were immersed in sodium hydroxide solution with a fixed concentration of 6% for 24 h [20]. After alkaline treatment, the kenaf fibres were washed thoroughly with running water and dried in an oven at a temperature of 110 °C for 24 h.

#### 2.2.2. Silane Treatment

Surface treatment is then followed with silane coupling agent method. In this treatment, 0.5%, 1%, and 2%, respectively of APS (aminopropyltriethoxy silane) was dissolved in a solution which contained 70% methanol and 30% water. Next, the solution was stirred for 30 min. Then, the kenaf fibre which had already endured the alkaline treatment soaked in silane solution for 3 h and dried in an oven at a temperature of 110 °C for 24 h to remove all the fibre’s moisture content.

Samples have been classified depending on three different types of silane concentration, neat polymer, and untreated kenaf fibre as tabulated in Table 2.

#### 2.2.3. Composite Mixture

The kenaf fibre and polymer matrix were prepared using the law of mixture formula as per in Table 3. To obtain the composition of composites, weight of elements has been calculated using Equation (1).

Weight Percentage of Element, we × Weight of composites = Weight of elements(1)

#### 2.2.4. Extrusion of Filament

A twin screw extruder has been used to produce filament composites with different parameters as shown in Table 4.

**Figure 1:**
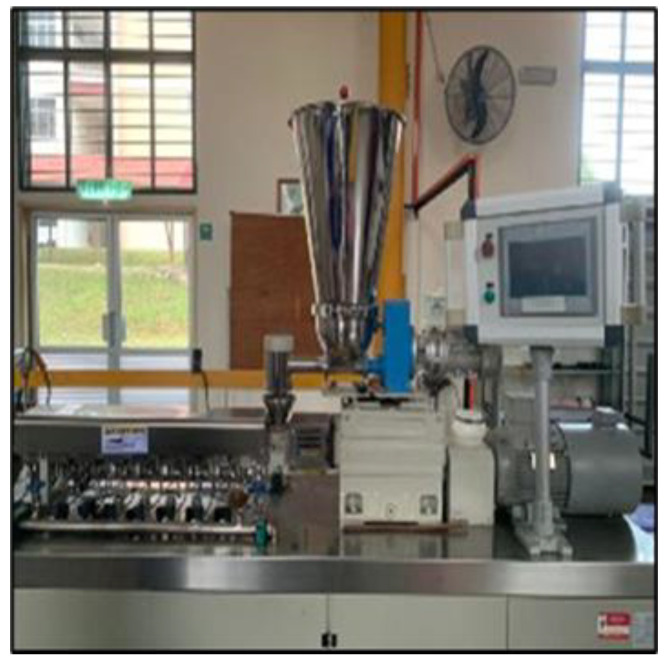
Twin screw extruder.

**Figure 2:**
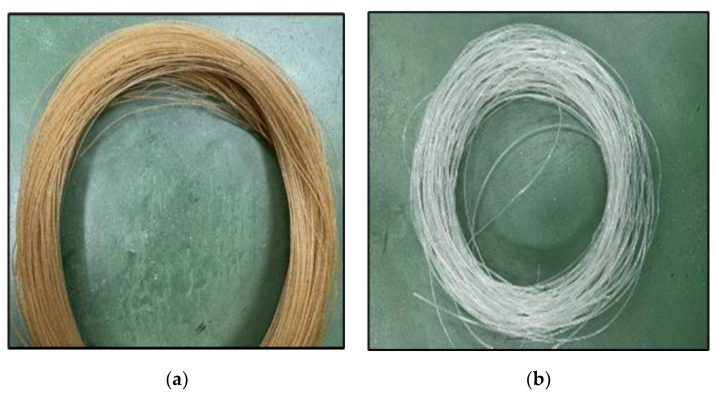
Kenaf fibre reinforced PLA composites: (**a**) Kenaf fibre PLA composites filament; (**b**) Neat polymer filament.

With a constant pulling speed of 25.5 rpm and constant filament size of 1.75 mm [34], the twin screw extruder is shown in Figure 1 while Figure 2 shows the biodegradable filament of kenaf fibre and neat PLA.

#### 2.2.5. Sample Extrusion

**Figure 3:**
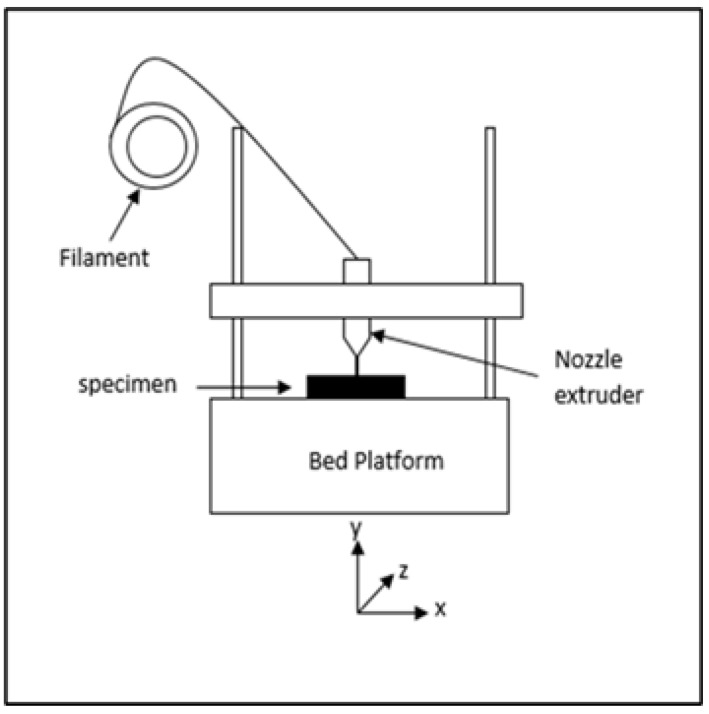
Schematic 3D printing process.

**Figure 4:**
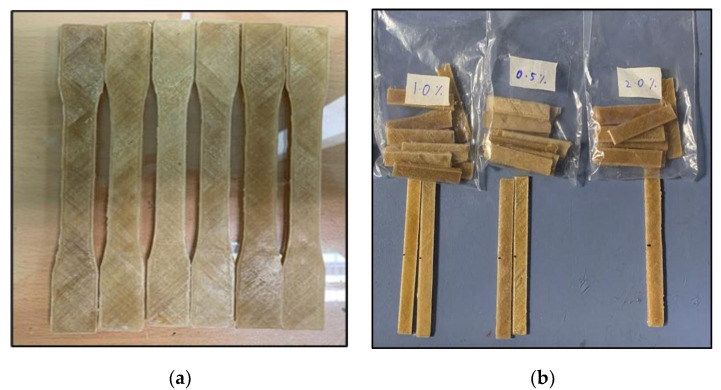
(**a**) Tensile specimens (**b**) Flexural specimens.

The sample had been extruded using Flashforge 3D printing as illustrated in Figure 3. Next, there were several parameters that needed to be considered such as the temperature of the nozzle, the temperature of the bed, and the percentage of infill. The parameter that had been set up where the solid infill is set to 100% in-line shape. The shell’s parameter is two layers, while the upper and bottom layer is three repeated numerical layers. Layer height was 0.18 mm, while first layer height is 0.27 mm. Next, nozzle temperature had been set up to 210 °C and bed temperature at 60 °C as PLA polymer does not require a high temperature. The printing speed also affected the performance of printed samples. In this printing process, the speed of the nozzle was 60 m/s while travel printing was 80 m/s. Figure 4 depicts the tensile and flexural specimen via 3D printing. 

## 3. Sample Characterisation

### 3.1. Mechanical Test

In order to evaluate the mechanical properties of the biodegradable composites, the tensile test was applied. Some of the properties that can be obtained after performing the tensile test include Young’s Modulus, maximum elongation, tensile strain, and yield stress. The sample size is in a “dog bone shape”, which is type 1 of the three listed types. For this research, the testing was carried out by following the ASTM D638 standard. By using this standard testing, the crosshead speed is 1 mm/min with a load cell of 5 kN with a span length of 50 mm. The tensile properties of composites were determined using the Universal Testing Machine model Instron 887, manufactured in Norwood, Massachusetts, United States.

The tensile strength of the single fibre can be calculated using Equation (2).

(2) $$\sigma =\frac{F}{A}$$

where, *σ* is the tensile strength of the fibre (Pa), *F* is the maximum force at break (N), and *A* is the area of the cross section (m^2^).

Using a three-point bending set up by following the ASTM D790 standard, the flexural test was conducted. Using this standard testing, the crosshead speed is 1 mm/min with a load cell of 5 kN. About five samples each from samples A, B, C, D, E, F, G, and H were taken and tested using the Universal Testing Machine model Instron 5585 manufactured in Norwood, Massachusetts, United States. The sample size is 100 × 10 × 3 mm following the ASTM standard with a span length of 50 mm.

The flexural test of the single fibre can be calculated using Equation (3).

(3) $$\sigma =\frac{3PL}{2bd^{2}}$$

where, *σ* is flexural strength of the fibre (Pa), *P* is maximum force at break (N), *L* is support span (mm), *b* is the width of the beam tested (mm), and *d* is the depth of the beam tested (mm).

### 3.2. Thermogravimetric Analysis (TGA)

The thermogravimetric analysis (TGA) was performed in order to obtain the degradation of the kenaf fibre under a high temperature before forming into composites. This analysis is conducted by using a machine from TA instruments and a filament specimen following the ASTM D3850 standard. The temperature rate was between 10 °C and 900 °C with a heating rate of 10 °C/min. The TGA was obtained using a Thermogravimetric Analyser located at Mettler-Toledo (M) Sdn. Bhd., Selangor, Malaysia.

### 3.3. Fourier Transform Infrared Spectrometry (FTIR)

The Fourier Transform Infrared Spectrometry (FTIR) was conducted using Jasco FT/IR-6100 (manufactured in the United States) on five different samples in an untreated powder state, at 0.5% silane, 1.0% silane, and 2.0% silane, in order to obtain the functional group for each different surface treatment. All spectra were recorded in the range of 4000 cm^−1^ to 400 cm^−1^.

### 3.4. Differential Scanning Calorimetry (DSC)

Differential Scanning Calorimetry (DSC) was performed on five sample filaments in a nitrogen atmosphere; neat polymer, untreated, 0.5% silane, 1.0% silane and 2.0% silane. The temperature range is 10 °C up to 300 °C, with a heating rate of 25 °C. The Differential Scanning Calorimetry (DSC) was obtained using DSC Q20 V24.11 Build 124.

### 3.5. Morphological Analysis

For this research, morphological studies were performed in detail on the fractured surface of the tensile test sample using a Scanning Electron Microscope (SEM). The five different samples taken from the tensile specimen were tested; neat polymer with untreated composites at 0.5% silane, 1.0% silane and 2.0% silane were taken in 2.5 wt % of fibre loading. The samples were coated with platinum to get a better result of resolution as it offers good electrical conductivity. The micrograph was obtained by using a JSM-6010PLUS/LV Scanning Electron Microscope (Jeol Ltd., Tokyo, Japan).

## 4. Results and Discussion

### 4.1. Mechanical Test

Tensile and flexural strength were performed by mechanical testing and measured their strength and Young’s Modulus [30].

Strength of composites might be influenced by several factors such as interfacial bonding. Good stress distribution could obtain good results [35].

Ververis et al. (2012) stated that tensile could be represented as one type of stress working in one direction (1-D). This testing could indicate whether the sample has good or bad in interfacial bonding. Maximum tensile strength, elastic modulus, and strain to failure can be obtained by enduring the tensile test [36].

**Figure 5:**
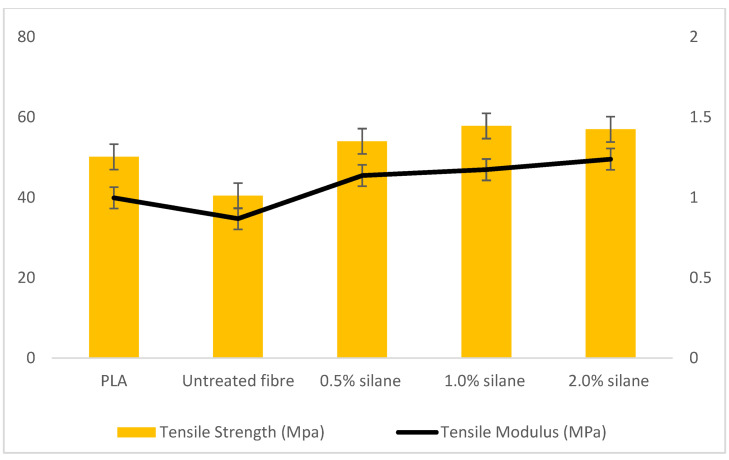
Results of composites tensile strength, MPa, and tensile modulus, MPa.

In this experiment, the data in Figure 5 was obtained with three different classes which were neat polymer (PLA), untreated kenaf fibre, and treated kenaf fibre. Treated kenaf fibre used the process of immersion in alkali and silane. Three different concentrations of silane solution were used: concentrations 0.5%, 1.0% and 2.0%. Asim et al. (2016) and Oushabi et al. (2017) stated that alkaline treatment could remove the impurity, lignin, and hemicellulose of kenaf fibre and enhance the interfacial bonding between two phases (fibre and polymer), but the parameters that need to be considered are the concentration of alkali itself and the time of immersion. This paper also claimed that by enduring alkali and silane treatment, a good result in tensile strength could be obtained as compared to the untreated fibre [7].

The tensile test was carried out following all the specific guidelines from ASTM D638 Standard. The graph bar above shows that the treated fibre indicates good strength as compared to the neat polymer and untreated fibre. It can be concluded that good mechanical properties were obtained due to surface treatment as compared to the untreated fibre [37]. Fibre orientation is not applicable in this experiment because the fibre used is in a powder state, known as isotropic, which means it doesn’t have a specific orientation involved.

Next, the treated group using different types of silane concentration obviously shows that 1.0% (57.85 MPa) of silane treatment is the strongest as compared to 0.5% (54.01 MPa) and 2.0% (56.99 MPa) silane concentration, respectively. The obtained results showed that a 1% concentration of silane gave the optimum tensile strength as compared to the others. It also showed that introducing the fibres to PLA plastic had improved the mechanical properties. These results revealed that the removal of lignin and hemicellulose by enduring silane treatment showed good interfacial bonding between the matrix and fibres. The efficient removal of impurities might be done by using a higher concentration of silane but might degrade the tensile strength due to rupture of the surface fibre and degradation of the fibre chemical content [7]. Nevertheless, if the concentration is lower, the impurities might not be removed perfectly, and the strength of the composites was also affected due to the hydrophilic property of the fibre. In previous research in which optimum concentration was studied, it was reported that lower concentrations might not work efficiently [6].

Lee (2009) experimented by varying the concentration of silane and concluded that approximately 1% created optimal strength and binding of composite as compared to 3% and 5% [3]. Yucheng Liu [5] investigated corn stalk fibre-reinforced polymer composites by using four different types of silane concentration. From all the testing performed, this paper concludes that 1% is the most optimal as compared to other silane concentrations, as a higher concentration might affect the surface and reduce the special characteristic of the fibre itself. Another paper by Yucheng Liu [1] also observed the different type of concentrations on the results of a mechanical test involving the tensile, as well as an impact test. 1% silane concentration treatment is optimal according to the mechanical test, as compared to other concentrations. This is because silane is an acidic liquid, and if the usage is high, it will corrode the original structure and strength of the fibre [1]. The variation of concentration, time and effect of the surface fibre has been discussed by R. Mahjoub [12]. Mahjoub et. al. (2014) also concluded in their paper that the higher the concentration and immersion time, the greater the decrement in the breaking strength of fibre.

In addition, by referring to the literature review, one might say that the condition of composites might also be affected during the manufacturing process. The difference between treated and untreated fibre composites might be due to the surface cleaning, as treated fibre promotes better adhesion bonding between the two phases and increases the strength of composites [2].

Flexural testing was done in order to determine the strength and the ability of the material to resist the deformation under loading before reaching the break point [38]. This technique to evaluate and obtain modulus elasticity in bending and flexural stress as the material was set up as supported beam under two supports with load applied at a point [39].

**Figure 6:**
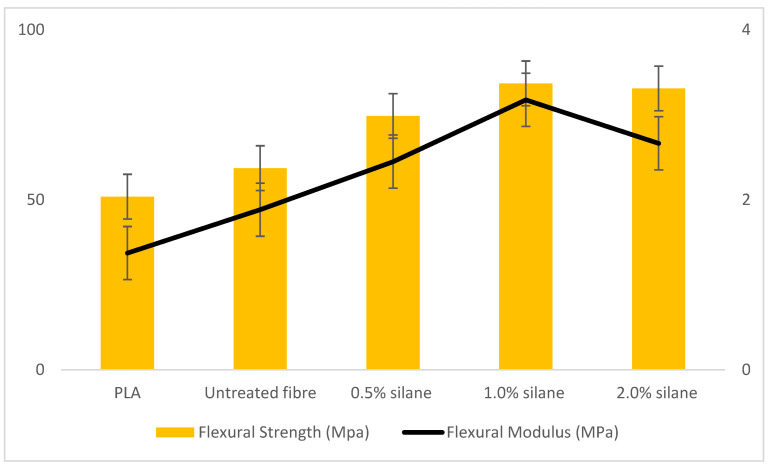
Results of composite flexural strength (MPa) and flexural modulus (MPa).

Flexural testing observes whether the composites could withstand bending load and deformation before they fail [36]. A flexural test was carried out following all the specific guidelines from the ASTM D790 standard. Three categories have been set up in this flexural test which are neat polymer, untreated, and treated fibre, using silane in different concentrations. The data in Figure 6 shows that 1.0% silane obtained high flexural strength (84.22 MPa), while the flexural modulus was the second highest as compared to 2.0% silane (82.74 MPa). Whereas the untreated composites achieved low strength (59.30 MPa) as compared to treated composites, but overall, PLA achieved the lowest strength(50.93 MPa). The treated fibre obtained good results, presumably due to the silane treatment which enhanced the interfacial bonding and had good dispersion stress while applying force.

Regarding the flexural modulus, 1.0% silane indicated a high modulus (3174.76 Pa) as compared to other composites and neat polymers. This proved that 1.0% silane treatment is the optimal concentration for kenaf fibre composites. The untreated polymer had low data, which may be due to low interaction linkage between the fibre and polymer, or poor dispersion of fibre towards matrix which leads to weak load transfer and may be due to voids during manufacturing of the composites as compared to treated composites [30,38]. It can be concluded that the higher the strength of composites, the stronger the bonding between two phases (reinforced and polymer matrix). The good interlocking composites can also be achieved by enduring the chemical treatment with the optimal concentration [36]. This concluded that a good surface treatment parameter process can lead to good strength and elasticity while performing any test.

**Figure 7:**
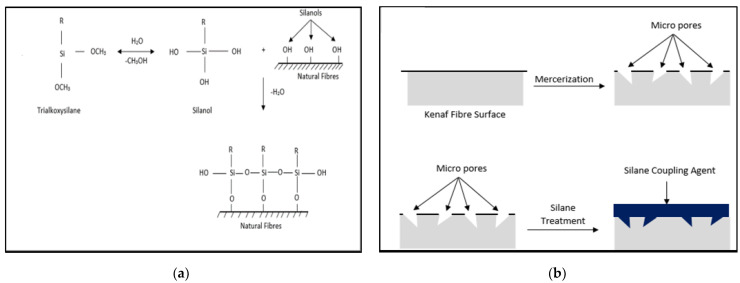
The overview reaction of surface modification (**a**) Silane reaction; (**b**) Mercerisation and silane treatment process.

From the results obtained, it was found that the treatment enhanced the tensile properties of the composite samples due to the interfacial adhesion between the fibres and matrix. Figure 7a indicates the molecular structure of trialkoxysilane, which acts as a silane coupling agent for the kenaf fibre. In the presence of water (H_2_O), the trialkoxysilane would develop the active agent of silanol in the reaction with the kenaf substrate. This silanol structure underwent a condensation process and naturally deposited on the kenaf surface to form a siloxane bond between the kenaf and silane coupling agent. This can result in a functional kenaf surface where the organofunctional groups can react with the PLA resin and produce better adhesion between the fibre and the matrix. Figure 7b illustrates how alkaline and silane treatment react towards natural fibre surfaces.

### 4.2. Thermogravimetric Analysis (TGA)

**Figure 8:**
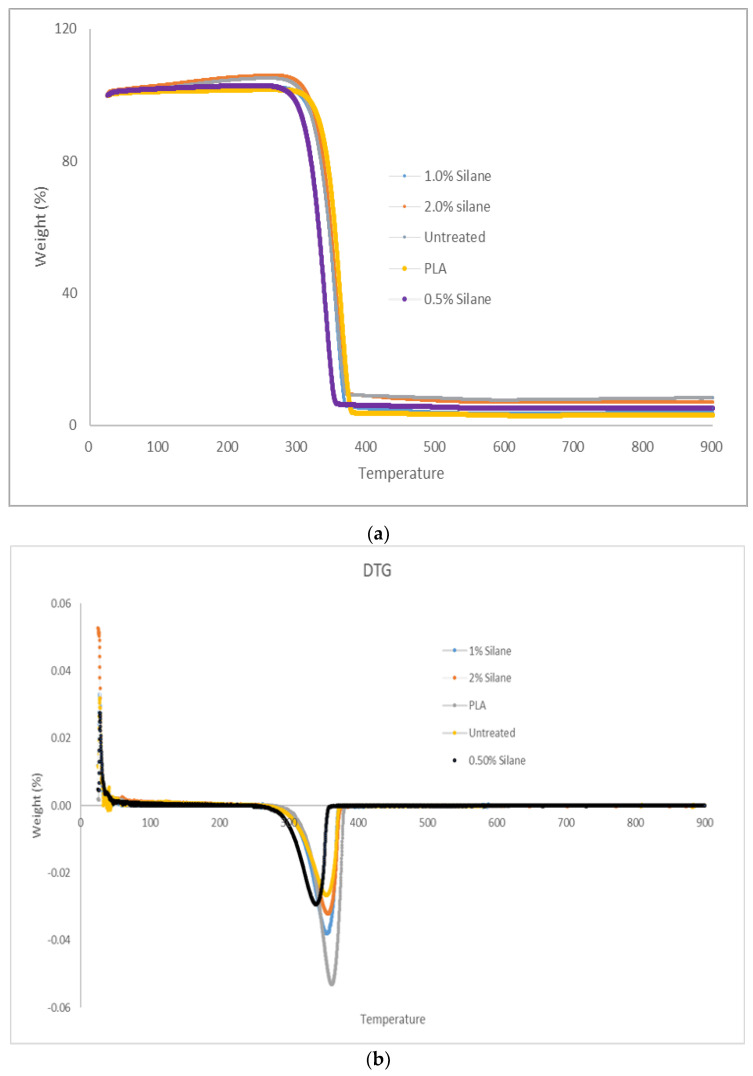
The results of composites (**a**) Thermogravimetric analysis (TGA); (**b**) Difference thermogravimetry (DTG).

Thermogravimetric analysis (TGA) and difference thermogravimetry (DTG) illustrated in Figure 8a,b in a nitrogen atmosphere is used to measure the thermal stability, thermal decomposition, and mass changes of PLA, untreated, and treated kenaf fibre composites. Due to properties of thermoplastics that allow them to be recycled and reused, thermal degradation is required to observe the degradation of composites at certain temperatures. This procedure can provide information on the composites capability to withstand high temperatures. TGA and DTG show the characteristic of composite degradation under nitrogen air. Five samples were conducted and started to degrade at certain temperatures.

Three phases of degradation had been stated by K. Krishna [40]. First, the degradation phase where the moisture content started to evaporate, followed by a second phase in which a high temperature was applied, the chemical content such as hemicellulose, cellulose, pectin and lignin was removed, and in the last stage, the final residue was below 10 wt %.

In TGA analysis for kenaf fibre-reinforced PLA composites, the first phase degradation occurs at 10–300 °C. In this phase, fibres start to lose moisture as it is evaporated [41] and the weight loss in this stage is currently below 9 wt % [42]. At temperatures of 300 °C up to 400 °C is where the chemical content such as cellulose, hemicellulose, pectin, and lignin in fibre slowly degraded [40,41,42]. This is due to the high temperature applied towards kenaf fibre. Hemicellulose is the functional group that degraded first, followed by cellulose and lignin; cellulose is more stable than hemicellulose [40]. From the observation, 1.0% silane composites needed a high temperature to break the functional group. Lastly, the final phase was the remaining of composites after applying the maximum temperature. The maximum temperature applied was 90 °C, leaving the weight residue below 10 wt %. From the obtained results, the main degradation temperature of the structural component of kenaf fibre was 300–400 °C. This means that it is suitable for the 3D printing application as the process temperature is around 160 °C to 210 °C. The process temperature depends on the types of filament. It is recommended to set the melting temperature of the material a bit higher to ensure all the filament is fully melted during the extrusion process [34].

Char residue is produced when cellulose is decomposed at a high temperature. This is evident in Figure 8a,b, where the percentage of char residue in treated fibre is lower in comparison to untreated fibre because the presence of lignin and cellulose which is not removed from untreated fibre. The result of TGA parallels previous researchers’ findings which concluded that thermal stability may be improved through alkali treatment. In addition to that, the reduction in char residue content is a result of decrement in the formation of carbonaceous char.

### 4.3. Chemical Analysis by Using Fourier Infrared Spectrometry (FTIR)

**Figure 9:**
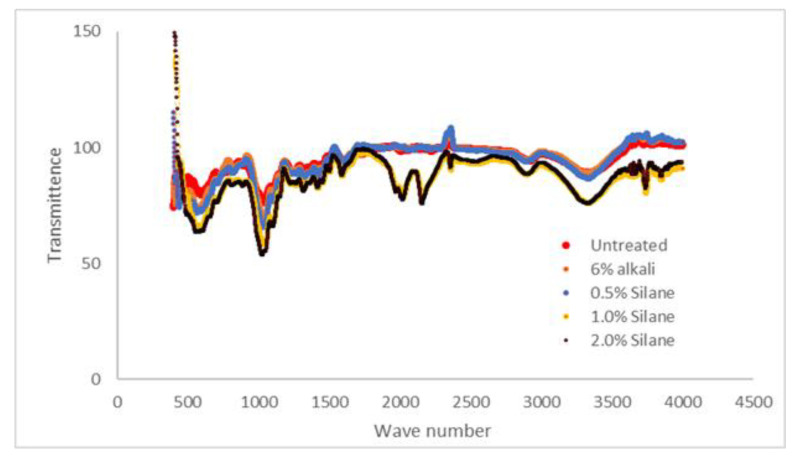
Fourier Transform Infrared Spectrometry (FTIR).

Figure 9 and Table 5 show the FTIR spectra for neat polymer, untreated, and treated kenaf fibre-reinforced PLA composites. Cellulose, hemicellulose, and lignin are components that were detected in the FTIR spectrum [43]. For example, the group of C–O stretching from lignin indicated clearly at peaks of 1000–1300 cm^−1^ [7,43]. From the data, the untreated fibre shows fibre lignin at a peak of 1035 cm^−1^, and 6% NaOH at a peak of 1030 cm^−1^; for fibre that endured silane treatment (0.5%, 1.0%, 2.0%), the peak was at a range of 1029–1030 cm^−1^. The decreasing trend of the wave number shows that the lignin was removed from the fibre.

Based on theoretical peak data, CH and -CH_2_, which are hemicellulose and cellulose, exist at a range of 2858 cm^−1^ to 2926 cm^−1^. Untreated fibre has shown that hemicellulose and cellulose exist at peaks of 2924 cm^−1^ and 3308 cm^−1^, respectively. From the data we can see that fibres that treated with the alkaline treatment and 0.5% silane have no marked difference for hemicellulose, which is 2899 cm^−1^, but for cellulose, NaOH and 1.0% silane indicated no difference where the peak is at 3334 cm^−1^.

### 4.4. Differential Scanning Calorimetry (DSC)

**Figure 10:**
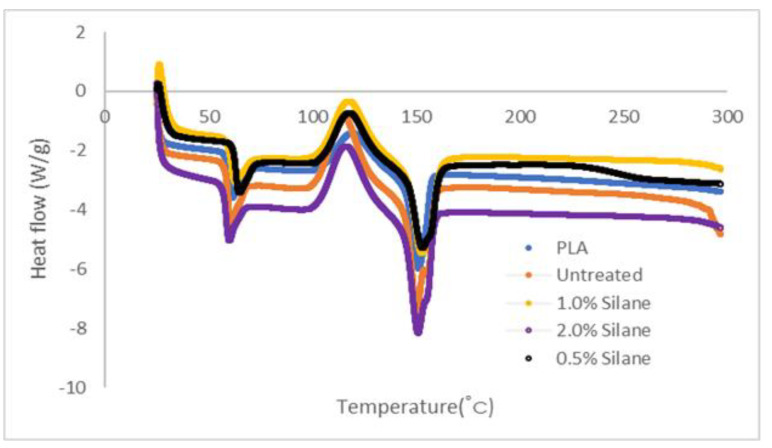
Differential Scanning Calorimetry (DSC).

Figure 10 and Table 6 compare the temperature value of neat polymer, untreated, and treated kenaf fibre-reinforced PLA composites. The values have been tabulated into Table 6. The terms exothermic and endothermic are the main keys in graph reading. Exothermic peak for the PLA polymer is the crystallisation temperature, while endothermic peak is at the melting temperature of 151.23 °C and the degradation temperature of 298.75 °C [44].

The neat PLA DSC curves and those of PLA composites with untreated, 0.5 % silane, 1.0% silane, and 2.0% silane of treated kenaf fibre composites indicate the glass transition temperature of PLA (58.69 °C), untreated (58.06 °C), at 0.5% silane (61.89 °C), 1.0% (59.32 °C), and 2.0% silane (57.04 °C). By setting the neat polymer crystal temperature benchmark (120.12 °C), the thermography shows that in untreated 0.5% silane, 1.0% silane, and 2.0% silane fibre composites, the PLA polymer chains did not crystallise completely, as illustrated in the temperature peaks of kenaf fibre composites. The crystallisation temperatures that had been achieved from the data are 115.36 °C, 118.67 °C, 118.29 °C, and 116.88 °C, respectively. The melting temperature is significantly different between composites and neat PLA. By referring to the table, the melting temperature of untreated (149.61 °C), 0.5% silane (152.35 °C), 1.0% silane (152.87 °C) and 2.0% silane (150.94 °C). The difference between each data for melting temperature of PLA composites is in the range of 1 °C, indicating that kenaf fibre does not interfere with the processing temperature. The temperature of degradation for each of the parameters circulated between 291.75 °C and 29.75 °C. This occurred because the PLA itself degraded the polymer chain and there was a loss of hydrogen elements after the rupture happened. The thermal properties of the filament are one of the important factors to be determined before the printing process. This is due to the need to add thermal properties of the filament as an input parameter during the printing process. If the thermal energy is not adequate during the process, it will affect the quality of the samples and lower the mechanical properties. The exact melting temperature will also help the uniform distribution between fibre and polymer in the extrusion process and avoid the problem of a clogged nozzle [34].

### 4.5. Morphological Analysis

**Figure 11:**
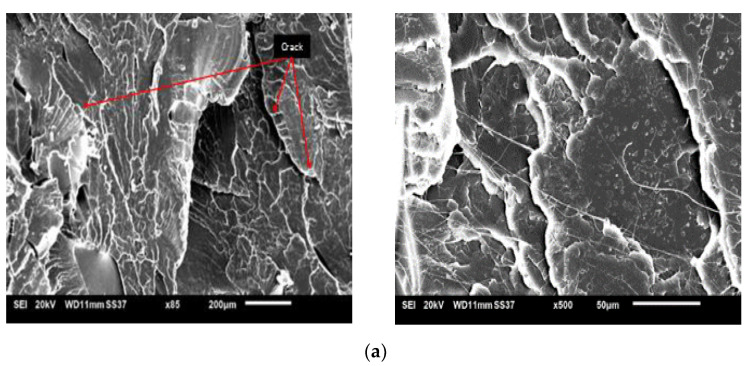

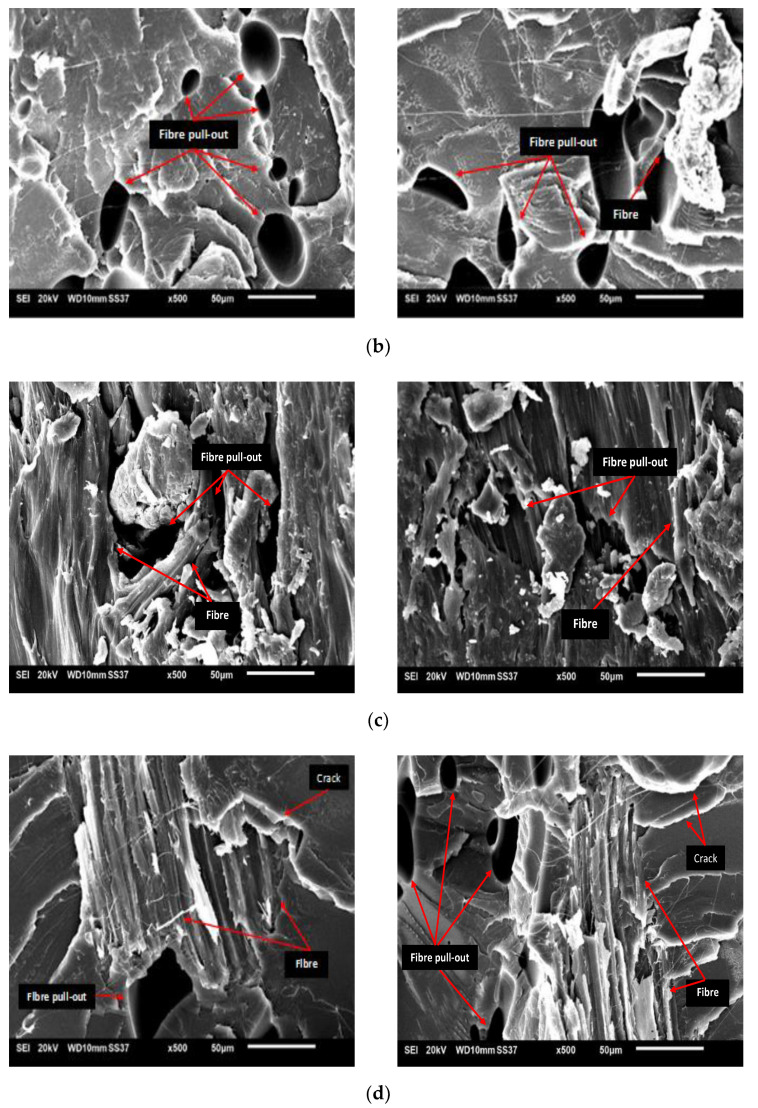

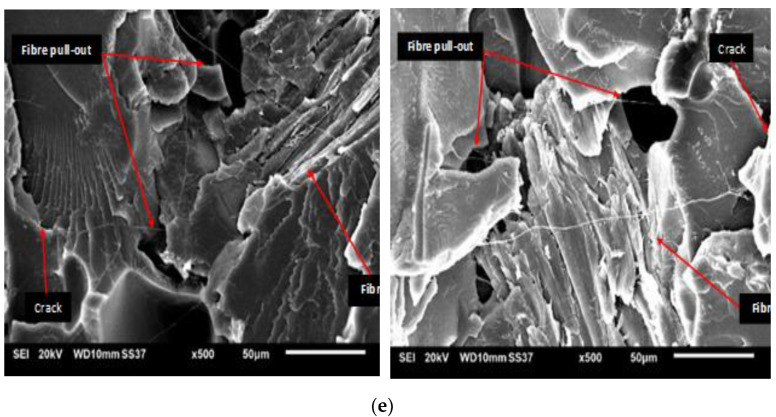
Scanning Electron micrograph (**a**) PLA; (**b**) Untreated fibre; (**c**) 0.5% silane; (**d**) 1.0% silane; (**e**) 2.0% silane.

Scanning Electron Microscope (SEM) is a method to review the detail of a kenaf fibre-reinforced PLA surface. This test was performed to check the adhesion bonding between treated and untreated fibre composites after enduring the tensile test. Figure 11 shows the SEM micrographs (×500) of the kenaf fibre composites.

Figure 11a indicates the smooth neat polymer surface defects. From the observations, it has been proven that PLA resins have a ductile manner as compared to untreated and treated fibres, which are brittle. Figure 11b of untreated kenaf clearly shows that fibre pull-out occurred, and as proven, a hole exists on the surface of the composites. This case happened due to the impurity of untreated fibre that get affected because of weak interfacial bonding between natural fibres and polymers (2). As a result, when load was applied, the fibre could not withstand and pulled off from the grip of the matrix. As a comparison to the treated fibre shown in Figure 11c–e, with three different concentrations of silane, it can be seen that there is still fibre left. Figure 11c, which was treated with 0.5% of silane shows impurities still exist on the composite surface. In conclusion, 0.5% silane cannot remove impurities as well as 1.0% and 2.0% silane. The fibre left on the surface proves that the bonding between the fibre and the polymer occurs perfectly; thereby the dispersion of load towards composites is distributed equally. Therefore, 1.0% silane and 2.0% silane of treated fibre achieved a high result in strength as compared to other composites, but results show that 1.0% silane is the most optimal concentration for silane.

**Figure 12:**
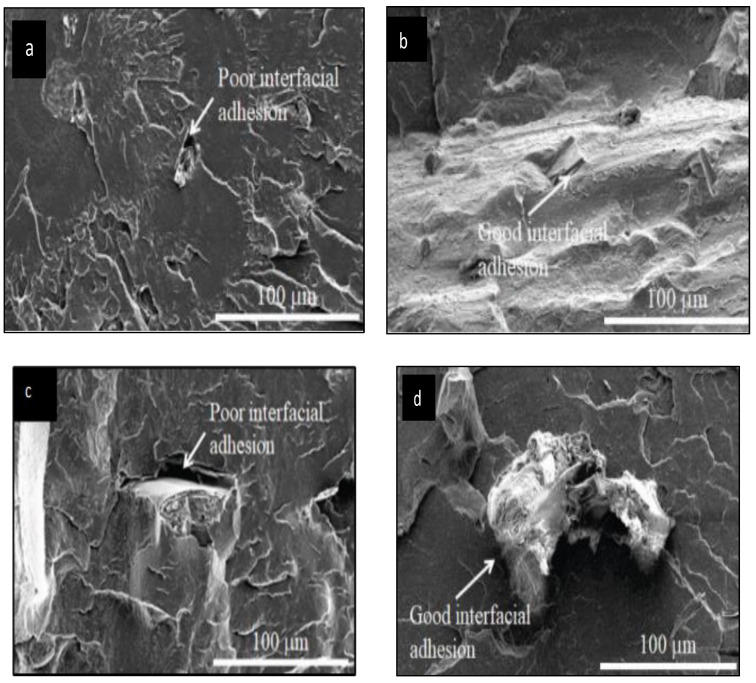
Scanning Electron Micrograph (**a**) Untreated wood PLA (**b**) Treated wood PLA (**c**) Untreated wood PLA (**d**) Treated wood PLA [32].

Torrado et al. (2014) stated that the use of silane in addition to surface modification after the alkaline treatment improved some of the minor factors such as dispersion and adhesion of the reinforcement and polymer matrix [45], and the SEM micrograph in Figure 12 has been compared with Figure 11 in silane treatment towards the fibre surface. In past experiments, Petchwattana et al. [32] stated that a silane coupling agent enhanced the bonding interaction between hydrophilic wood flour and hydrophobic PLA polymers [46,47]. Figure 12 shows the bonding between fibre and PLA polymer, untreated and treated with a silane coupling agent. Figure 12a,c show poor interfacial adhesion between the untreated fibre and polymer, while Figure 12b,d show good interfacial bonding with treated fibre.

## 5. Conclusions

For many years, researchers have tried to determine the correct method to produce high-performance materials by using natural fibres as reinforcement agents. Many methods have been discovered in combining reinforcing agents with a polymer matrix, and this paper is focuses on mixing kenaf fibre with thermoplastic PLA, and observing the mechanical and physical properties of the composites. FDM is one of the additive technologies that has many advantages, such as rapid production, good finishing, ability to generate many shapes with complicated geometries and dimensions, and low cost. This research focuses on the effect of using chemical treatment on kenaf fibre and analysis thereof by performing mechanical and physical tests. In this paper, 2.5 wt % of kenaf fibre was mixed with a PLA polymer by using a twin screw extruder, and a 3D printer filament was extruded. Five different parameters were produced, including neat polymer (PLA with 0 wt % of fibre), untreated kenaf fibre composites, and three different silane treatment parameters. Samples that were printed following ASTM standards were used to performed the tests, and data has been collected. Based on data observation, 1.0% silane concentration after being treated with a 6% alkali solution indicated that this parameter can enhance the interfacial bonding between two phases, which occur due to removal of chemical content in the natural fibre itself such as cellulose, hemicellulose, and lignin. This experiment also proves that applying a higher silane concentration can lead to fibre damage. For example, 2.0% silane is the highest concentration of silane, and the strength data shows that 2.0% silane is lower than a concentration of 1.0 %. Meanwhile, untreated natural fibre composites obtain the lowest strength due to poor interfacial bonding because the stress cannot distribute equally on the surface, unlike treated composites. In a nutshell, for natural fibre modifying composites, the most crucial element that needs to be considered is the optimal concentration of silane for natural fibre surface treatment, as it creates appropriate bonding to achieve a high strength for application development.

## Figures and Tables

**Table 1:** Fibre characteristic values for tensile strength (MPa), Young’s Modulus (GPa), elongation (%) and density (g/cm^3^) [11,12,13,14,15].

Fibre	Tensile Strength (MPa)	Young’s Modulus (GPa)	Elongation (%)	Density (g/cm^3^)
Cotton	287–800	5.5–12.6	3.0–10.0	1.5–1.6
Jute	393–800	10.0–30.0	1.16–1.8	1.3–1.6
Flax	345–1500	27.6	1.2–3.2	1.4–1.5
Hemp	550–900	70.0	1.6–4.0	1.47–1.48
Sisal	400–700	9.0–38.0	2.0–14	1.33–1.5
E-glass	2000–3500	70.0–73.0	2.5–3.4	2.50–2.55
Carbon (standard)	3400–4800	230–425	1.4–1.8	1.4–1.78
Kenaf	930	53.0	1.6	1.2–1.45
PALF	170–1627	60.0–82.5	1.6–2.4	1.56

**Table 2:** Sample classification.

Parameter	Explanation
PLA	Neat polymer
Untreated	Untreated kenaf fibre composites
0.5% silane	0.5 wt % silane concentration + 6% alkali concentration kenaf fibre composites
1.0% silane	1.0 wt % silane concentration + 6% alkali concentration kenaf fibre composites
2.0% silane	2.0 wt % silane concentration + 6% alkali concentration kenaf fibre composites

**Table 3:** Composition of composites.

Samples	Weight of Composites (g)	Weight of Fibre (g) 2.5 wt %	Weight of Matrix (g) 97.5 wt %
All samples	500	12.5	487.5

**Table 4:** Parameter of extrusion.

Samples	Melting Temperature (°C)	Screw Speed (rpm)
PLA	210	25
Untreated fibre	190	29
0.5% silane	204	25
1.0% silane	204	25
2.0% silane	204	25

**Table 5:** Parameter of extrusion.

	Untreated	6% NaOH	0.5% Silane	1.0% Silane	2.0% Silane
Lignin	1035	1030	1029	1030	1029
Hemicellulose CH	2924 (2936–2916)	2899	2899	2902	2902
Cellulose CH_2_	3308	3334	3333	3334	3335
Absorption of H_2_O	1597	1499	1420	1421	1421
Hydroxyl Group -OH	3400–3200	3400–3200	3400–3200	3400–3200	3400–3200
Si-C Stretching Bond Silane	8958	826.3	813.8	900–700 (no peak)	900–700 (no peak)
Stretching N-H Vibration	3328–3250 (SYM_STR)	3400–3332 (SYM_STR)	-	-	-
Ester Carbonyl Group C=O	1727	1592	1593	1593	1593

**Table 6:** Differential Scanning Calorimetry (DSC) data.

	Thermal Properties		
Parameter	Tg (°C)	Tcc (°C)	T_m_ (°C)
PLA	58.69	120.12	151.23
Untreated kenaf/PLA	58.06	115.36	149.61
0.5% silane kenaf/PLA	61.89	118.67	152.35
1.0% silane kenaf/PLA	59.32	118.29	152.87
2.0% silane kenaf/PLA	57.04	116.88	150.94

## Data Availability

The data presented in this study are available on request from the corresponding author.
